# ‘Not at the diagnosis point’: Dealing with contradiction in autism assessment teams

**DOI:** 10.1016/j.socscimed.2020.113462

**Published:** 2021-01

**Authors:** Jennie Hayes, Rose McCabe, Tamsin Ford, Daisy Parker, Ginny Russell

**Affiliations:** a University of Exeter, College of Medicine and Health, EX1 2LU, UK; b School of Health Sciences, City, University of London, Myddelton Street Building, 1 Myddelton Street, London, EC1R 1UW, UK; c University of Cambridge, Department of Psychiatry, Douglas House, 18b Trumpington Road, Cambridge, CB2 2AH, UK

**Keywords:** Diagnosis, Uncertainty, Autism, Sociology of diagnosis, Discourse, Narrative, UK

## Abstract

Social science literature has documented how the concept of diagnosis can be seen as an interactive process, imbued with uncertainty and contradiction, which undermines a straightforward notion of diagnosis as a way to identify underlying biological problems that cause disease. We contribute to this body of work by examining the process of resolving contradiction in autism diagnosis for adults and adolescents. Autism is a useful case study as diagnosis can be a complex and protracted process due to the heterogeneity of symptoms and the necessity to interpret behaviours that may be ambiguous. We audio-recorded and transcribed 18 specialist clinical assessment meetings in four teams in England, covering 88 cases in two adult, one child and one adolescent (14+) setting. We undertook a qualitative analysis of discursive processes and narrative case-building structure utilised by clinicians to counteract contradiction.We identified a three-part interactional pattern which allows clinicians to forward evidence for and against a diagnosis, facilitates their collaborative decision-making process and enables them to build a plausible narrative which accounts for the diagnostic decision. Pragmatism was found to operate as a strategy to help assign diagnosis within a condition which, diagnostically, is permeated by uncertainty and contradiction. Resolution of contradiction from different aspects of the assessment serves to create a narratively-coherent, intelligible clinical entity that is autism.

## Highlights

• Diagnosis of autism in adults and young people can be complex and contradictory.
• Discursive analysis examined how teams deliver a coherent diagnostic narrative.
• An interactional pattern in team decision-making helps to resolve contradiction.
• Considering social outcomes is used as a strategy to help assign diagnosis.
• This decision-making process contributes to how we understand autism.

## 1 Introduction

Diagnosis is traditionally thought of as a way to identify underlying biological problems that cause disease. Scholars working in the field of sociology have problematized this, claiming diagnosis is a social process that involves multiple actors and is context specific (see Blaxter, 1978; Brown, 1995; Jutel, 2013; 2009; Jutel and Nettleton, 2011). Some argue that uncertainty is central to medical practice (e.g. Beresford, 2006; Bursztajn et al., 1986) because diagnosis is an act of interpretation and involves transposing clinical research to the idiosyncrasies of the individual patient (Tanenbaum, 1993). Atkinson (1995) argues for a detailed analysis of how clinicians locate the sources and nature of doubt, and express them discursively, arguing that this approach will further understanding about how medical knowledge is organised socially and produced through discourse (Atkinson, 1995).

There is a surge of interest in the conceptualisation of Autism Spectrum Disorder, henceforth ‘autism’, as a diagnostic category (e.g. Evans, 2013; Eyal et al., 2010; Fitzgerald, 2017; Nadesan, 2005; Silverman, 2013). Despite this, there remain few empirical studies which examine in detail how autism diagnosis actually comes about in practice. In previous work we examined how clinicians diagnosing autism produce objective accounts through their situated practices, and perform diagnosis as an act of interpretation, affect and evaluation (Hayes et al., 2020). This article contributes further to the sociology of diagnosis literature by using a discursive approach to examine narrative case-building in clinical assessment teams. Autism diagnosis for both adults and children can be a complex and protracted process due to heterogeneity of symptoms, the necessity of interpreting behaviours that may be ambiguous, and a lack of clinical biomarkers (Klin et al., 2000; Vllasaliu et al., 2016). It is therefore an ideal case study to explore how clinicians, in their role as diagnosticians, manage these complicating factors.

## 2 Background

### 2.1 Diagnostic uncertainty, contradiction and pragmatism

Uncertainty and contradiction have long been important topics for medical sociology in relation to clinical training (e.g. Atkinson, 1984; Fox, 1957; Timmermans and Angell, 2001); pharmaceuticals and neuroscience (e.g. Fitzgerald, 2014; McGoey, 2009; Pickersgill, 2011); classification, patient/doctor interaction and diagnostic decision-making (e.g. Bowker and Star, 1999; Bursztajn et al., 1986; Hedgecoe, 2003; Star, 1989; Zayts et al., 2016); medically unexplained or contested diagnoses (e.g Armentor, 2017; Dumit, 2006; Jutel, 2011; Marks et al., 2016); as well as in science more broadly (e.g. Campbell, 1985; Edwards, 1999; Fochler and Sigl, 2018; Pinch, 2012; Shackley and Wynne, 1996; Star, 2009).

Atkinson (1984) argues that rather than assuming uncertainty as a ‘taken-for-granted’ concept within medical sociology, certainty and uncertainty reflect different attitudes which may co-exist together. The ‘moral certainty’ of practical reasoning, experience or routine, is adopted in day-to-day clinical practice; and the uncertainty of theoretical discourse is the knowledge of the laboratory (Atkinson, 1995). Freidson (1970) argues that clinicians must believe in the efficacy of their actions rather than attend to research that identifies the unreliability of diagnosis, or is rooted in uncertain findings, for example. These factors lead the clinician to be a pragmatist focussed on results and with an emphasis on emotional experience, rather than general concepts or probabilities. In this way the clinician comes to rely on the ‘authority of his *(sic)* own senses’ (Freidson, 1970, p.170).

It could be argued, therefore, that the science of medicine is filtered through the hands of the experienced clinician, which can outweigh any research-based evidence (Timmermans and Berg, 2003). A focus on experience may enable the clinician to find pragmatic certainty within a context of conflicting evidence and inconclusive tests.

Gardner et al. (2011) examined how clinicians make sense of potential contradiction in diagnosis of chest pain. Drawing from the work of Mol (2002), the authors argue that ontology is fluid and transitory, and assembled through interaction between entities in the clinic. By following the trajectory of one patient from GP to cardiologist, they demonstrate how particular practices assemble information such as family background, the impact of exercise and the results of an ECG, that then enables the patient's chest pain to become intelligible as a clinical entity (Gardner et al., 2011). Different (and potentially contradictory) accounts from these interactions are consolidated through discrediting one source of information (the ECG test) enabling the clinician to reframe the condition with a sense of coherence. The effect of this ‘patching together’ (Gardner et al., 2011, p. 849) of multiple accounts is to reify the notion into a ‘singular coherent body’ (Gardner et al., 2011, p. 848). As with Latimer's (2013) ethnographic study of dysmorphology, clinical judgement within the wider picture is privileged over any single test.

To conclude, Brown (1987) suggests that diagnosis in psychiatry is inherently contradictory due to the multiple goals of diagnosis, and the acceptance of a task (diagnosis) which is not entirely in the design of the psychiatric profession. Multiple external agendas may conflict with patient-care objectives, placing the clinician in a potentially ambiguous situation. A pragmatic approach to diagnosis may enable clinicians to deal with the institutionally-induced competing demands of diagnosis. For example, in psychiatric diagnosis, an interview study identified that psychiatrists engage in ‘psychiatric workarounds’ such as negotiating diagnoses with patients or fudging codes on paperwork (Whooley, 2010).

### 2.2 Diagnosis of autism

Autism is diagnosed when persistent patterns of difficulty in social communication and interaction, combined with restricted and repetitive patterns of behaviour, interests or activities are judged to cause significant impairment in functioning (APA, 2013). The most commonly used assessment measure for both adults and children is the Autism Diagnostic Observation Schedule (ADOS) (Lord et al., 2000), alongside a clinical interview such as the Autism Diagnostic Interview-Revised (ADI-R) (Le Couteur et al., 2003). The ADOS is an activity-based semi-structured standardised observation tool whereby the person performs a number of communication and interaction tasks, and is then scored on a range of behaviours such as emphatic or emotional gestures and overall quality of rapport. First-hand reports from the patient, and their informants (usually a family member) are utilised, and the impact of associated impairment on the individual and the family is considered. Diagnosis is undertaken, therefore, primarily on the basis of observation and informant accounts.

Deciding where the diagnostic threshold lies can be problematic as autism symptoms are distributed as a continuum extending into the general population (Constantino 2011; Constantino and Charman, 2016). Hollin (2017a, 2017b) argues that the work of cognitive researchers in the 1980s/1990s embedded the concept of heterogeneity as core to the definition of autism (Hollin, 2017b). Rather than seeking to erase uncertainty, Hollin argues, researchers have effectively centralised it within the meaning of autism itself, creating a condition that is ‘determined by its indeterminacy’ (Hollin, 2017b, p617). The implications for clinical practice are that clinicians are compelled to work within a system that requires a categorical diagnosis within a condition constructed as an ‘uncertain entity’ (Hollin, 2017a, p209), with the resulting potential for ambiguity, uncertainty and contradiction, particularly around those that may be considered ‘threshold’ cases.

### 2.3 Narrative and autism diagnosis

There is a significant body of work which demonstrates how narrative is shaped and purposed in medical settings (see Ainsworth-Vaughn, 1998; Atkinson, 1995; Bosk, 1992; Byrne and Long, 1976; Hunter, 1991; Mattingly, 1991; 1998; Mischler, 1984; D. Silverman, 1987) including conversation analytic studies into patient-doctor communication (e.g. Heritage and Maynard, 2011; 2006; Maynard and Heritage, 2005; Pilnick et al., 2009).

Classic studies of narrative by Labov and Waletzky (Labov, 1972; Labov and Waletzky, 1967) argue that, at its most basic, a narrative takes the form of two clauses, ‘temporally ordered’ (Labov, 1972). Labov found that narrative story-telling formed a basic six-part structure: abstract, orientation, complicating action, evaluation, resolution and coda (Labov, 1972). Atkinson (1995) demonstrates how, in a haematology clinic, clinicians follow a similar narrative case-building process to establish a diagnostic outcome, drawing on the temporal facts of a case which can serve to ‘scaffold’ uncertainty within a framework of uncontested assertions.

Particularly relevant to our study is a body of work exploring autism assessment in the US (see Maynard and Turowetz, 2019; Turowetz, 2015a, 2015b; Turowetz and Maynard, 2019, 2016). Maynard and Turowetz claim that there is a universality to the narrative of diagnostic practice across social settings and historical periods and this takes a structural form in four parts: preface (an introductory narrative); possible stories (either tendency – reporting a general propensity towards particular patient behaviour; or instantiation – reporting a single instance); typifications (categorical assertions which include diagnostic upshots); and story recipiency (how the story is received, supported or facilitated by others in the room) (Maynard and Turowetz, 2017; Turowetz and Maynard, 2017).

The researchers demonstrate how clinicians collaborate in assessment to build a narrative case together, which enables ‘interactional progressivity’ (Maynard and Turowetz, 2017, p. 265) towards diagnosis. Rather than simply organising and documenting evidence, narrative practices ‘play a constitutive part’ (Turowetz and Maynard, 2017, p. 20) in aligning symptoms to disease categories, in this case, shaping autism as they diagnose it in practice.

### 2.4 The current study

Other than Maynard and Turowetz's study, there remains very little work observing how clinicians discuss autism diagnosis together and none that we know of related specifically to narrative case-building in adolescent and adult diagnosis. The current study takes a narrative focus to examine how clinicians make diagnostic decisions together in the light of ambiguity or contradiction. It builds on previous work by including analysis of diagnosis of adults and young people over the age of 14; siting the study in a UK context where the passing of legislation between 2009 and 2011 led to the development of adult diagnostic services; and specifically looking at how clinicians deal with contradiction in their narrative accounts.

## 3 Data and method

### 3.1 Sample and recruitment

We purposively sampled teams that specialised in autism assessment and who held regular assessment meetings (two in adult assessment; one with children; and one with adolescents (14+)). Recruitment was undertaken from an open call to a list of clinical contacts drawn from the internet and via the National Institute for Health Research (NIHR) Clinical Research Network. All teams were located in England and were National Health Service (NHS) providers. Three teams were multi-disciplinary with specialists from different disciplines (including psychiatry, clinical and educational psychology, speech and language therapy, nursing, social work, and occupational therapy). One team (adults) was single-disciplinary and primarily included clinical psychologists and assistant psychologists. Written informed consent was obtained from all participants, and ethical approval was granted by the University of Exeter ethics committee and by the Health Research Authority (HRA). Patients and families were not present at any meeting.

### 3.2 Data collection

We observed and audio-recorded over 19 hours of data from 18 autism assessment team meetings, covering discussion of 88 cases. The purpose of the meetings was to discuss specific cases after assessment or referral. Audio data were transcribed, anonymised and entered into Nvivo for data management and initial coding. We used the concept of ‘information power’ (Malterud et al., 2016) to assess when we had adequate data to meet the aims of the study. This involved consideration of quality of dialogue, analysis strategy, use of established theory and sample specificity.

### 3.3 Analysis

We took a discursive psychology (DP) approach to enable an investigation of how participants' talk created a case for or against diagnosis. A DP approach treats discourse as social action, rather than as an insight to people's motivations, emotions or underlying cognition (Edwards and Potter, 1992; Wiggins, 2017). Reports are constructed as factual by way of a variety of discursive techniques (Edwards and Potter, 1992) or devices (Wiggins, 2017) such as narrative structure, footing shifts, category entitlements and consensus/corroboration. DP attempts to work with participants' own ‘sense-making’ practices (Wiggins, 2017) in that it examines how participants orient to the meaning of each utterance – how they make sense of each other in talk.

The process of analysis followed guidance as outlined by Wiggins (2017) and drew from the theoretical framing of DP by Edwards and Potter (1992). We first identified sequential phases of the discussions, for example, openings, presenting problems, diagnostic recommendations and closing sequences, enabling us to identify patterns in the data and focus on particular features to be analysed. As our interest was in diagnostic decision-making, we then searched for points where participants came to disagreement or consensus about assessment and what happened before and after these instances. At these points we identified social actions (e.g. requests for information, asking and replying to questions, seeking clarification) and examined the presence of discursive techniques as outlined by Wiggins (2017) and Edwards and Potter (1992) to consider how reports were constructed as factual. We became interested in how clinicians resolved potential contradiction in their deliberations. Detailed transcripts were made using Jefferson transcription to enable analysis of the turn-by-turn detail of conversation (Jefferson, 2004). We examined the specific sequential positioning of expressions of contradiction and their resolution. Finally we returned to the complete data set and checked for instances across the data, further refining the analysis. The developing analysis was conducted by the first author, presented regularly at data analysis sessions, and discussed with co-authors throughout to develop and challenge emerging ideas and to develop consensus.

### 3.4 Note on terminology

We use the term ‘clinician’ to encompass all healthcare participants in this study, defined as members of a registered health profession involved in direct patient care. We use the term ‘patient’ to describe all people attending autism assessment, however, we understand the limitations and medical nature of this term.

## 4 Analysis and results

Of the 88 cases recorded, 51 were diagnosis specific. Of these 51 cases, 43 included discussion of potential or diagnosed co-conditions; 24 were child or adolescent cases and 27 were adults. Twenty-five cases were female, 25 were male and one was transgender. There were 27 cases with a diagnostic outcome declared in the meeting (15 female, 12 male; 11 adults, 16 children or adolescents) where it was possible to track when evidence discussed was contrary to the final diagnostic decision. For the purposes of this study, we only included those cases with a diagnostic outcome. In four cases the diagnostic decision made was in contradiction to the ADOS result (three cases were under-threshold on ADOS and diagnosed with autism: one was over-threshold and not diagnosed). Two of those cases - one adult and one adolescent (from 14+ team) - are discussed in detail here.

### 4.1 Three-part interactional pattern

**Fig. 1:**
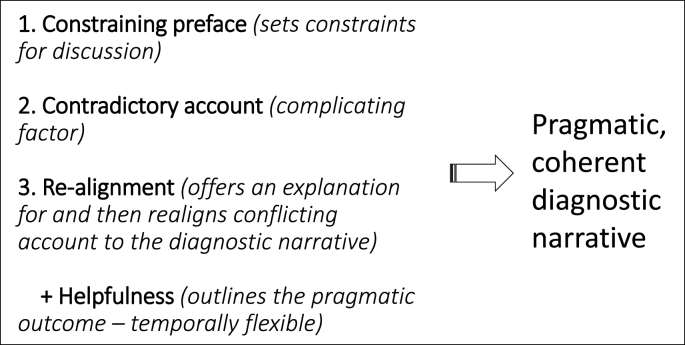
Three-part interactional pattern in autism diagnostic discussions.

Whilst specific details of team discussions varied according to the purpose of the case discussion and the participants attending, we found a recurrent three-part interactional pattern utilised to account for and explain contradictory evidence, illustrated in Fig. 1. First, a ‘constraining’ preface formed part of the ‘opening’ of the discussion and served to set the agenda for discussion and flag up any assessment issues. Second, contradictory accounts were given which constituted evidence considered not to be in alignment with the current diagnostic trajectory. Third, there was a realignment of that contradictory account to align with the eventual diagnostic decision, enabling resolution and production of a coherent diagnostic narrative. In addition, clinicians frequently invoked how ‘helpful’ diagnosis would be for the patient or family. This temporally flexible device of helpfulness was accommodated at different times in the interaction, and provided a rationale for the realigned account whilst demonstrating orientation to patient-care objectives.

The three-part interactional pattern was present in 19 of the 27 cases with a diagnostic outcome (see Table 1). Of the remaining 8 cases, 6 were considered to be straightforward with no contradictory accounts, and 2 included contradictory evidence but had a ‘neutral’, rather than constraining preface. A constraining preface was present therefore in 25 cases and contradictory accounts were present in 21. In 23 cases, there was an invocation of how helpful diagnosis may be for the patient or family. In cases where diagnosis was assigned, contradictory evidence was most commonly explained by ‘masking’ (n = 13): the development of strategies to compensate socially thereby making observation of autistic behaviours in assessment problematic.

**Table 1:** Three-part interactional pattern.

Case no	Age	Sex	ADOS result	Pattern	1. Preface	2. Contradictory evidence	3. Re-alignment	Helpfulness	Co-conditions	Outcome

**Adult team 1 (11 cases discussed in total, 1 with outcome)**										
2	51–60	Male	Under threshold (4)	Three part	Constraining	Under threshold on ADOS	Compensating^a^; under-reporting	Yes	Yes	Diagnosed ASC

**Adult team 2 (16 cases discussed in total, 10 with outcome)**										
12	26–35	Female	ADOS score not mentioned	No (straightforward)	Constraining	n/a	No contradictory evidence	No	Yes	Diagnosed ASC
13	36–50	Female	Under threshold (2)	Three part	Constraining	Under threshold on ADOS	Compensating	Yes	None mentioned	Diagnosed ASC
14	18–25	Male	Too difficult to score	Three part	Constraining	Traits; social difficulties; variable eye contact; delayed language development	BEDC^b^ (schizoid personality disorder)	Yes	Yes	Not diagnosed
15	51–60	Female	ADOS not mentioned	No (straightforward)	Constraining	n/a	No contradictory evidence	Yes	Yes	Diagnosed ASC
16	18–25	Female	Meets threshold (7)	No (straightforward)	Constraining	n/a	No contradictory evidence	Yes	None mentioned	Diagnosed ASC
19	26–35	Female	Under threshold (2)	No (straightforward)	Constraining	n/a	No contradictory evidence	No	None mentioned	Not diagnosed
21	36–50	Female	Under threshold (2)	Three part	Constraining	History and AQ10/RQ suggestive of ASC; repetitive behaviours	BEDC (OCD/ADHD/attachment)	Yes	Yes	Not diagnosed
22	36–50	Female	Meets threshold (9)	Three part	Constraining	Social interaction ok; lot of gesture	Compensating	Yes	Yes	Diagnosed ASC
25	26–35	Male	Under threshold (4)	Three part	Constraining	Special interests	BEDC (anxiety)	Yes	Yes	Not diagnosed
26	26–35	Male	Meets threshold (11)	No (straightforward)	Constraining	n/a	No contradictory evidence	Yes	None mentioned	Diagnosed ASC

**C&YP Team 1 (20 cases discussed in total, 12 with outcome**										
28	5–10	Female	Under threshold	Two part	Neutral	Parental report	Over-reporting	Yes	Yes	Not diagnosed
31	14–18	Female	Meets threshold	Three part	Constraining	Over threshold on ADOS; history and symptoms suggestive of ASC	BEDC (depression/neglect)	Yes	Yes	Not diagnosed
32	14–18	Male	Under threshold	Three part	Constraining	Language difficulties; traits; repetitive behaviours	BEDC (neglect/language difficulties)	Yes	Yes	Not diagnosed
34	14–18	Female	Not discussed (didn't do full ADOS)	Two part	Neutral	Good eye contact, insight, facial expression; no language difficulties	Compensating (gender)	Yes	Yes	Diagnosed ASC
36	14–18	Male	Meets threshold	Three part	Constraining	Good social manner, eye contact, social play	Compensating	Yes	Yes	Diagnosed ASC
37	11–13	Male	Meets threshold (9)	Three part	Constraining	Other prof and one parent believes not-ASC	ADOS is clearly above threshold; some compensating	Yes	Yes	Diagnosed ASC
38	5–10	Male	Meets threshold	No (straightforward)	Constraining	n/a	No contradictory evidence	Yes	Yes	Diagnosed ASC
40	5–10	Female	Meets threshold	Three part	Constraining	Non verbals under threshold on 3di; some gesture	Compensating; under-reporting	Yes	Yes	Diagnosed ASC
41	11–13	Female	Meets threshold	Three part	Constraining	Absence of observed repetitive behaviours	Subsequent report of repetitive behaviours; no other explanation	No	Yes	Diagnosed ASC
42	5–10	Male	Meets threshold (9)	Three part	Constraining	Eye contact, non-verbals, emotion recognition OK; other profs believe not-ASC	Compensating	Yes	Yes	Diagnosed ASC
44	5–10	Male	Meets threshold	Three part	Constraining	Gets on well socially, lots of friends; language within normal limits	Parents under reporting; compensating	Yes	Yes	Diagnosed ASC
47	5–10	Male	Meets threshold	Three part	Constraining	Good non-verbals, eye contact, language, pretend play/conversation; no echolalia	Compensating	Yes	Yes	Diagnosed ASC

**C&YP Team 2 (4 cases discussed, all outcomed)**										
48	14–18	Female	Meets threshold (not yet scored up)	Three part	Constraining	Early to talk; no mannerisms; uncertain about stereotypical/repetitive language	High 3di scores; ‘enough there'	Yes	Yes	Diagnosed ASC
49	5–10	Male	Meets threshold (not yet scored up)	Three part	Constraining	Good at recognising emotion, social/creative play; some gesture; socially motivated	Compensating	Yes	Yes	Diagnosed ASC
50	14–18	Female	Under threshold	Three part	Constraining	Under threshold on ADOS; good gestures, rapport, eye contact, facial expressions, imagination, building conversation	Compensating (gender); complicated by anxiety	Yes	Yes	Diagnosed ASC
51	14–18	Female	Meets threshold (not yet scored up)	Three part	Constraining	Good insight, gesture; some emotion recognition, roleplay/creativity	Compensating	No	Yes	Diagnosed ASC

a Compensating includes: learned behaviours, taught strategies, training (eg in presentation skills), masking and camoflauging; putting on a show.

b BEDC Better explained by different condition.

### 4.2 Case 13: Teresa, age 45

Teresa, a 45-year-old woman, is discussed by a Consultant Psychiatrist, Catherine, and the Team Manager, Jo, who is an Approved Mental Health Practitioner and experienced diagnostician. Catherine has conducted a clinical interview, and Jo an ADOS. The case discussion begins with a discussion to identify the patient being discussed. This is followed by Catherine's prefacing statement.

#### 4.2.1 Constraining preface

#13 Excerpt 1

**Figure:**
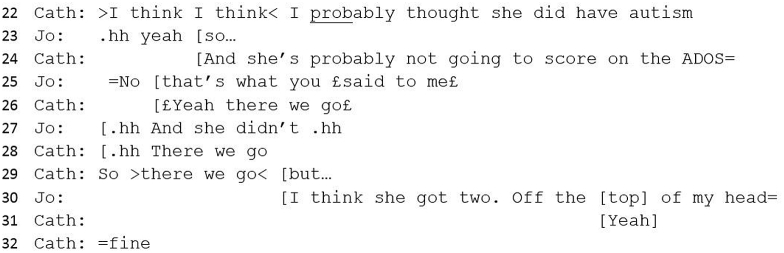

Catherine formulates the case (lines 22–24) and announces the potential outcome. Catherine's formulation sets the agenda: the patient has autism but this will not be apparent on the ADOS result. Catherine presents this potential contradiction as normative: not scoring and being autistic is not an unlikely outcome, perhaps drawing on shared knowledge that the ADOS tool lacks sensitivity - the ability of a test to correctly identify those with the condition - when diagnosing women and girls. Whilst hedging (‘probably’, ‘I think’) might represent uncertainty about the outcome or her memory of it, hedging allows space for disagreement without conflict and can be withdrawn or amended depending on Jo's response (Wiggins, 2017). Jo's response to Catherine's assertion is to agree, both with the statement that is what Catherine thought, and, then, with the ADOS result, although not necessarily with the assertion that Teresa does have autism. However, this interaction serves to strengthen Catherine's prefacing statement as it corroborates her predicted assessment, and her receipt of this is one that serves to reinforce the accuracy of her view (‘there we go’) and express her certainty.

During the following exchange the ADOS score is introduced, which aligns with Catherine's formulation, in that a score of two would be considered to be consistent with ‘not scoring’, i.e. under-threshold for diagnosis. The lack of precision and apparent lack of preparation to report the score (line 30) inoculates against any potential claims that Jo might be invested in the ADOS score (Wiggins, 2017) and serves to downplay its importance in the discussion. Jo's minimisation of the ADOS score, therefore, aligns with Catherine's initial declarative statement.

The constraining preface sets up the stories that follow (Turowetz and Maynard, 2017), flags pertinent issues and allows the teller to project the forthcoming story (Jefferson, 1978). In this study, the preface does this by including an implicit or explicit prediction or judgement about the outcome or suggesting a problematizing factor. The preface therefore sets the agenda for the resulting discussion, and potentially undermines alternative versions or topics. We do not mean by this that there is intention by the opening speaker to constrain, rather, interactionally, it serves a ‘shaping’ role as it takes the form of a statement against which other evidence must align or contradict: contradicting is generally more difficult in interaction.

#### 4.2.2 + Helpfulness (temporally flexible)

Between lines 33 and 45 Jo provides a tendency story – a general propensity to a type of behaviour (Maynard and Turowetz, 2017) (‘she did tend to do that going off on a tangent on something but she was a bit sort of quite rigid too’) which she checks with Catherine (‘did you find that with her?); but this is not supported by specific instances of autistic-like behaviours. Jo then goes on to describe a particular issue for Teresa.

#13 Excerpt 2

**Figure:**
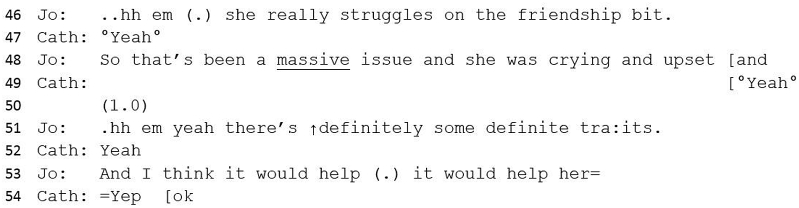

Jo uses intensifiers (‘really’, ‘massive’) which serves to upgrade an earlier assessment of Jo's (‘a bit sort of’) from minimised and tentative to significant. The objective description of difficult emotions (line 48) further upgrades Jo's assessment by illustrating Teresa's distress about her difficulties as genuine. Jo's statement that Teresa has ‘traits’ has the effect of more definitively supporting Catherine's diagnostic statement, particularly with the repeated use of ‘definite’, which bolsters the evidence to support a case for diagnosis.

Jo completes her turn by introducing a functional element to the potential diagnosis (‘it would help her’), making a pragmatic argument in support of diagnosis. Whilst there is no detailed discussion about how the diagnosis might be helpful, this can be seen as an account towards diagnosis and enables ‘interactional progressivity’ (Maynard and Turowetz, 2017): not evidence as such but a meaningful way to create justification for a decision.

However, in the next turn, Jo introduces a contradictory account, or complicating action (Labov, 1972), which would explain the earlier contradiction of a low-scoring ADOS. Unlike this earlier contradiction (lines 24 and 30), which is neatly incorporated into a diagnostic narrative (because Catherine has predicted a low-scoring ADOS), this second complication impedes a straightforward conclusion to the case discussion.

#### 4.2.3 Contradictory account

#13 Excerpt 3

**Figure:**
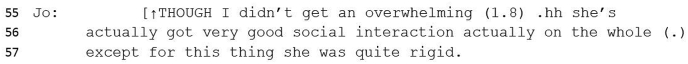

‘Though’ suggests a forthcoming contrastive element (Maynard and Turowetz, 2017, p. 265) – a statement against diagnosis in contrast to the trajectory of the meeting, whilst enabling Jo to give an implicit explanation for the low ADOS score. Jo embodies her own experience of meeting Teresa through her first-person declaration of how this felt (‘not overwhelming’) and tentatively queries the evidence for a diagnosis by citing ‘good social interaction’. A significant pause here suggests some difficulty in expressing this turn, perhaps due to disaffiliation with the general narrative towards diagnosis. Good social interaction would, generally, be an argument against diagnosis, as diagnosis requires ‘persistent impairment in reciprocal social communication and social interaction’ (APA, 2013). This utterance, therefore, should problematize a diagnosis of autism, however, Teresa's behaviour has already been accounted for as autistic (from line 22) and therefore, this statement is couched within a context of evidence towards rather than evidence against. Jo immediately counters her ‘good social interaction’ assessment with a contrast term (‘except for’) and then introduces a behaviour that would support a diagnosis – rigidity – suggesting an ambivalence toward a diagnosis.

#### 4.2.4 Re-alignment with diagnosis

Catherine responds.

#13 Excerpt 4

**Figure:**
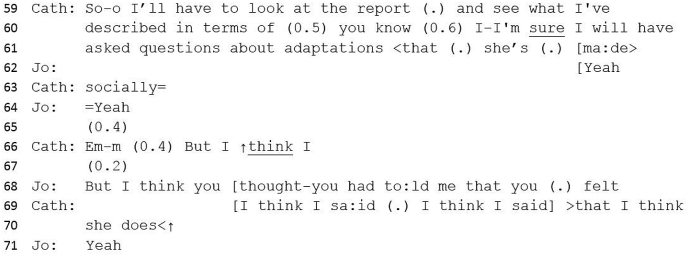

Catherine's so-prefaced response attends to Jo's problematizing factor, that of good social interaction, and offers a potential explanation of ‘adaptations’ for Teresa's social skills. With reference to her report and assessment, Catherine implies that Teresa's behaviour could look like good social interaction but is in fact adapted behaviour (masking) (line 61). Catherine appears to prepare to provide a further contrastive element (‘but’) (line 66); Jo, however, continues this utterance (line 68) which is completed by Catherine. Here Catherine (with Jo's corroboration) has again drawn a line under contradiction, not by reporting different evidence, but by re-asserting her clinical judgement (‘I think she does’).

Between lines 71 and 95, a colleague is tasked with finding Catherine's report. While they wait, Jo and Catherine discuss Teresa's family context. On finding the report, several tendency stories are cited by both Jo and Catherine related to Teresa's special interests (Maynard and Turowetz, 2017) and then there is a diagnostic upshot or resolution.

#13 Excerpt 5

**Figure:**
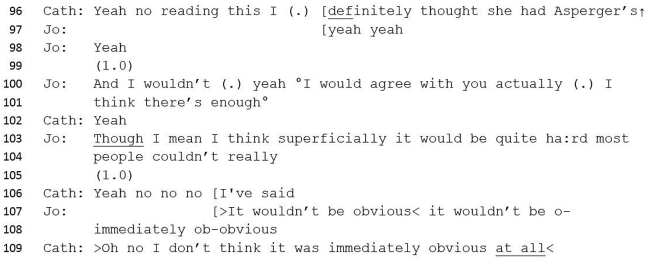

Catherine's rereading of her own report enables her to reassert her initial impression (line 96) with Jo's agreement taking place in line 100. Jo suggests that most people would not be able to identify autism in Teresa, and Catherine agrees. The interaction here serves both to index their professional expertise (lines 103–109) and to preserve it by accounting for why they too initially had some trouble ascertaining Teresa's behaviours as ‘autism’ (lines 107–108). This sequence serves to fit Teresa into a category of autism despite some behaviours that would not align with this diagnosis. Catherine and Jo successfully realign Teresa's ‘non-autistic’ behaviour within a category of autism by suggesting she has adapted her social difficulties to the extent that her social interaction appears to be very good, even though she still has underlying autism. Following this section, Jo relates an instantiation story which illustrates Teresa’s rigid behaviour in assessment, thereby further supporting a diagnosis. Further tendency stories (special interests, problems at work and difficulties with relationships) follow before a final diagnostic agreement.

#13 Excerpt 6

**Figure:**
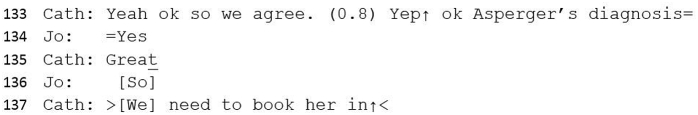

To summarise this case, clinicians can be seen to engage in a number of interactional devices to create a coherent diagnostic narrative for Teresa: setting the agenda for the discussion through a constraining opening preface; realigning contradictory accounts by discrediting one account (ADOS score) and invoking specialist understandings of autistic behaviours (adaptations); and making sense of the diagnosis by invoking the helpfulness of a potential diagnosis. In this way the potential uncertainty that might have been indicated by Teresa's good social interaction is managed by a realigned narrative around masking, adaptation and utility of a diagnosis, eliminating any expressed ambivalence towards the diagnosis.

### 4.3 Case 50: Gabrielle, age 15

Gabrielle is a 15-year-old girl who has been assessed by two Clinical Psychologists, Fatima and Carol; and a Consultant Psychiatrist, Maria. The psychologists, who have undertaken the ADOS, identify her behaviours as relating primarily to anxiety, and score Gabrielle as under-threshold (suggests not-autistic). The psychiatrist has conducted a clinical interview and has assessed Gabrielle as autistic. Bob is a visiting student doctor. After a brief discussion about the weather, Maria opens the case discussion.

#### 4.3.1 Constraining preface

#50 Excerpt 1

**Figure:**
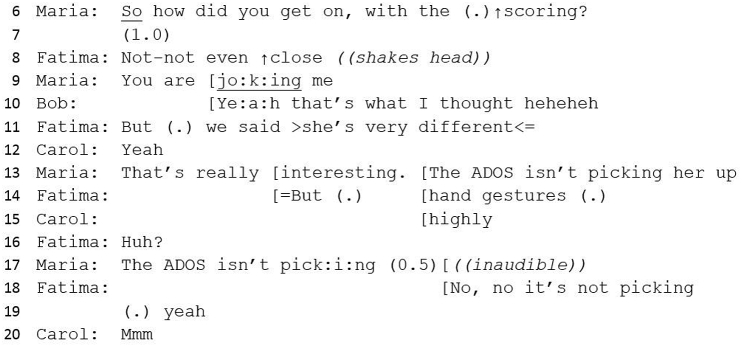

Maria begins the discussion with a request for information (line 6) which appears to invite an extended telling. Fatima's ‘headline’ response, therefore, (line 8) seems inapposite. Maria's follow-up response appears to orient to this by pursuing further telling and her surprised response to Fatima's negative score (line 9) sets the agenda for discussion as it signals her difference of view. The responses to line 9 illustrate that this statement is not received passively. Bob aligns with Maria's surprise; and Fatima's response (‘but we said she's very different’), in contrast to her utterance in line 8, offers a concession to the possibility of an autism diagnosis whilst maintaining positive relations with her colleague through suggesting the reasonableness of Maria's position prior to hearing the ADOS score. Through overlapping talk, as Fatima and Carol attempt to describe the reasoning behind the low score, Maria evaluates the contradiction with a disagreement term, suggesting the ADOS isn't ‘picking her up’, which is repeated by Fatima (lines 13–18), exhibiting further concession to Maria. This utterance locates responsibility for the contradiction in a failure of the diagnostic tool, rather than in the clinicians' role, treating the tool itself as an active agent (Anspach, 1988). It also refers explicitly to a commonly understood shortcoming of the ADOS amongst clinicians: that the ‘true positive rate’, or sensitivity, of the tool is inadequate in identifying women and girls with autism, leading to a diagnostic gender bias (see Lai et al., 2015; Loomes et al., 2017; Van Wijngaarden-Cremers et al., 2014). Fatima begins with a further disagreement in line 14, relative to Maria's position, which is continued in line 23 below. Overall, there is both disagreement and agreement taking place at this stage.

This opening sequence contains three aspects of our proposed pattern, thereby serving a comprehensive constraining function: preface, contradictory account (low ADOS score) and initial evaluation leading to re-alignment to diagnosis (inadequacy of ADOS tool). As with Teresa, it is striking that, even without any detailed narrative content (either instantiation or tendency stories), there is already an interactional ‘pull’ towards diagnosis at this early stage. It can also be seen that this narrative structure provides a social function in maintaining a collegial atmosphere whilst there are differences of opinion (Turowetz and Maynard, 2017). Maria's expression of the ADOS as an active agent in ‘not picking her up’, serves to distance the psychologists from their role in the ADOS result. Together this short interaction provides what we term a ‘constraining preface’, ensuring that participants immediately understand the key issues that need to be resolved. This is contrary to some types of case discussion, where an extended narrative, including an introduction to the patient, problem presentation, patient history and a chronology of events, is related prior to any clear evaluation of the difficulty (Anspach, 1988; Atkinson, 1995).

The psychologists go on to provide justification for the low ADOS score by outlining factors which would conflict with an autism diagnosis.

#### 4.3.2 Contradictory account

#50 Excerpt 2

**Figure:**
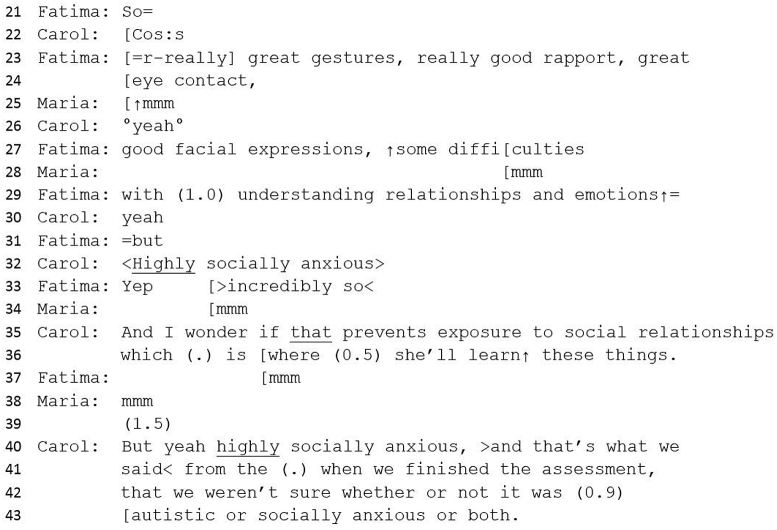

Fatima uses intensifiers (‘really great’) to describe behaviours not considered to be aligned with autism (lines 23–7), thereby bolstering her own position related to the ADOS score. These contradictory accounts are then contrasted with a minimised assessment (‘some’) of difficulties with understanding relationships and emotions (lines 27–29). On balance this utterance foregrounds non-autistic behaviours, and provides an account for the low score in the ADOS assessment, whilst allowing some opportunity for discussion of autistic-like behaviours. The contrast term (‘but’) is introduced and the turn is completed by Carol who interjects with their key finding: that Gabrielle is ‘highly socially anxious’, upgraded and corroborated by Fatima with, ‘yeah, incredibly so’. Carol's consequent explanation suggests that Gabrielle's anxiety may be impacting her socially. Her repetition of the anxiety assessment strengthens the weight of this view by invoking a corroborative agreement with her colleague (‘that's what we said’). Carol expresses uncertainty (‘we weren't sure’) which sidesteps a direct disagreement with Maria's assessment and instead, utilises uncertainty as an interactional resource (Pilnick and Zayts, 2014) by allowing space for renegotiation of the outcome. The potential for a diagnosis of *both* autism and anxiety offers a concession to Maria whilst maintaining the integrity of the psychologists' ADOS assessment. Whilst hedging and expressions of uncertainty may be used in part because Carol actually *is* uncertain, uncertainty markers also work to keep discussion open and flowing whilst potentially challenging clinicians who have more experience or higher status roles.

There follows a series of questions from Maria (e.g. ‘what about her quality of insight into relationships?‘) and an extensive overview of the clinical interview with Gabrielle's mother, which includes a series of reported stories (both instantiation and tendency) from Gabrielle's childhood, including typifying components towards an autism assessment (e.g. ‘she's not socially curious').

#### 4.3.3 + Helpfulness

During the case discussion, the team considers Gabrielle's preference for a diagnosis and how it might help her:

#50 Excerpt 3

**Figure:**
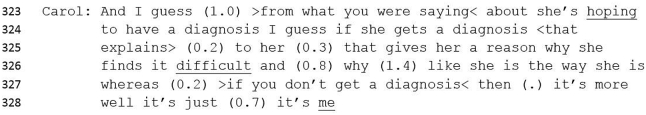

Here Carol tentatively speculates on the benefit for Gabrielle of receiving a diagnostic label, as an explanation for why she finds ‘it’ difficult. This interjection, prior to a diagnostic decision, provides further justification for a potential positive outcome, in that the diagnosis would make meaning for Teresa and offer her an explanation for her difficulties as well as allowing her (or indeed anyone with a diagnosis) to attribute their difficulties to a medical condition, rather than a personal failing.

#### 4.3.4 Re-alignment with diagnosis

After further discussion about the source and manifestation of Gabrielle's anxiety, Carol offers a formulation:

#50 Excerpt 4

**Figure:**
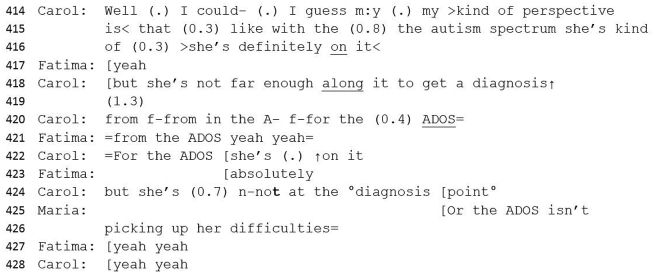

Carol accounts for her assessment by categorising Gabrielle as ‘on the spectrum’ - and therefore presumably diagnosable - and immediately contrasts this by suggesting instead that Gabrielle may be sub-threshold (‘but … not far enough along it’) (line 418). This contradictory account is then clarified, with a footing shift from ‘my kind of perspective’ to ‘from the ADOS’, which serves again to lay the difficulty with the ADOS and shift responsibility away from the clinician. The frequent pauses and hesitations suggest trouble (Jefferson, 1989) or the management of psychological business (Wiggins, 2017). Carol is attempting to summarise a diagnostic narrative inherent with conflict and contradiction at the same time as holding her turn to enable a full and nuanced account. She continues to assert ‘not autism’ but again offers a concession to a diagnosis by suggesting she is on the spectrum. Carol's complex account is again translated by Maria (lines 425–6) as an inadequacy of the ADOS, thereby providing an alternative account for the failure to reach ‘diagnosis point’. The psychologists concur with this analysis (lines 427–8). Here the first part of the re-alignment consolidates the inadequacy of the ADOS tool. Maria goes on to explain how this can happen:

#50 Excerpt 5

**Figure:**
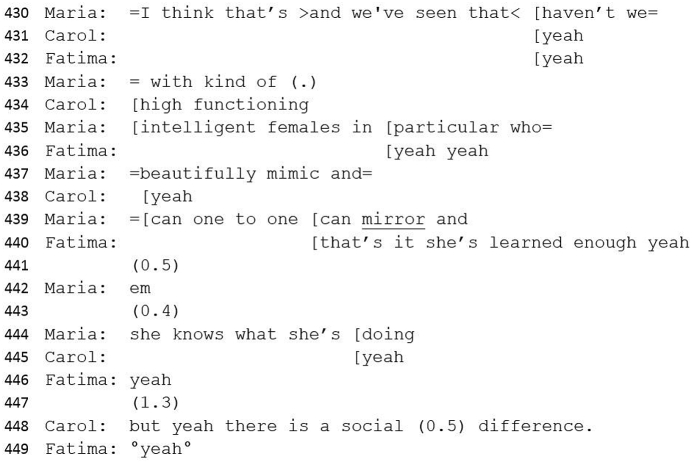

The second and explanatory part of the re-alignment compares Gabrielle to other ('high functioning, intelligent, female') patients who are able to mask symptoms of autism by mirroring and mimicking. Maria draws both psychologists into this concept of shared understanding (‘we've seen that haven't we’). Both recipients immediately demonstrate understanding (lines 431–2) and finally Carol suggests that despite Gabrielle's ability to mask symptoms, there is still a ‘social difference’ which would therefore contribute to an autism diagnosis (line 448).

Here the possibility of learned behaviour becomes a criteria for diagnosis and draws on a collective understanding that the ADOS has limitations when assessing women and girls as it is considered that women may have ‘subtler’ manifestations of social and communication difficulties (APA, 2013) which may not then show on the ADOS assessment. Fluctuations of disagreement, agreement and ambivalence culminate, via this re-alignment, in consensus.

## 5 Discussion

The study shows how clinicians bring together potential contradictory accounts to create a coherent narrative which then explains particular behaviours as autistic. We can see that contradiction caused by factors that might disrupt the idea of a ‘typical picture’ (Star, 1989, p. 73), such as strengths in social interaction; or the absence of others, such as repetitive behaviours or interests, are resolved by drawing on clinicians' assumed knowledge. These suppositions are embedded in their clinical, practical understanding of what autism does (or does not) look like, and are collectively drawn on and understood, virtually unnoticed (Pickersgill, 2011). The resolution of contradiction is possible due to the certainty that there is a ‘thing’ called autism despite ongoing uncertainties about aetiology, shifting definitions and lack of specificity about its neurobiology (Fitzgerald, 2014).

Our proposed narrative framework (Fig. 1) demonstrates how the interpretation of behaviours that look like’ autism (or not) are managed, enabling allocation to the appropriate disease category and the realigning of contradictory evidence. These strategies circumvent the messiness of diagnosing a condition defined by indeterminacy and ambiguity, to meet the institutional purpose of categorising behaviours in order to ‘do diagnosis’.

As with Maynard and Turowetz (Maynard and Turowetz, 2017; Turowetz and Maynard, 2017) we found that the narrative frame enables an evidential case for diagnosis to be built and constructs the case as a subject for medical talk, establishing the boundaries of what is important. We note, as do these scholars, that clinicians use different story-types (e.g. instantiation and tendency stories) to build a case for diagnosis, however, we also have shown that the detail of these stories can be minimal and tacit to the extent that understanding is shared or implied without being overtly stated. It is possible, for example, for team members to discuss the conflicting nature of ‘good social interaction’ without explicit detail of how this manifests in behaviours. It is also possible to produce a preface without an extended introductory narrative.

We consider our proposed narrative structure as complementary to Maynard and Turowetz's model and flexible in terms of their relationship, in that either pattern may work within the other in the pursuit of diagnostic resolution. Our model offers a specific detailed co-produced narrative framework by which contradictory accounts are managed and as a mechanism by which an impasse can be resolved in interaction. Evidence becomes an interactional product, in that the meaning of facts and evidence change with their use in discourse (Måseide, 2006), through the negotiation and evaluation of contradictory accounts towards a coherent diagnostic narrative.

Our work further adds to the sociology of diagnosis by demonstrating that clinicians consider an autism diagnosis, in some cases, as a pragmatic construct which enables them to offer patients and families a label which may provide understanding and (sometimes) access to support. This, in itself, is not a novel finding. Gill and Maynard (2012), examining the diagnostic delivery of a label of developmental disability, demonstrate how clinicians depict the diagnostic label as a tool for positive social outcomes for parents, which may enable them to achieve objectives (help and services) for their child. In autism assessment, other studies have examined the role of diagnosis in triggering access to services (e.g. Grinker, 2007; Rogers et al., 2016; Skellern et al., 2005) and drawn on the experiences of patients and families to explore how the delivery of a diagnosis provides a sense-making narrative, legitimization of experience, relief, exoneration from blame and a positive sense of identity (Crane et al., 2018; Hurlbutt and Chalmers, 2002; Jones et al., 2014; Molloy and Vasil, 2004; Punshon et al., 2009; Russell et al., 2019; Russell and Norwich, 2012). In our study, we have demonstrated how invoking ‘helpfulness’ – the pragmatic purpose of a diagnosis – can help to manage contradiction within the clinical team *prior to* diagnostic delivery, by providing a concrete, logical reason for the action. An invocation of ‘helpfulness’ – the potential for positive social outcomes – renders the diagnostic label as ‘relevant’, whether or not it sits within clear diagnostic boundaries (Gill and Maynard, 2012).

Further drawing from Gardner (2011), our analysis demonstrates that clinical entities are made intelligible by discrediting diagnostic accounts (e.g. the ADOS result) which enables the clinician not only to reframe the condition coherently but to provide an explanation for its dismissal that serves to contribute to the diagnostic narrative. With female patients in particular, contradictory accounts (a woman with good social skills but other autistic-type difficulties) are explained via masking, for example, which become incorporated into the diagnostic narrative as a clinical unified whole. This aligns with contemporary thinking that autistic women and girls may present better in social interaction than do men and boys because they are better able to mask their autistic behaviours. Diagnostic tools based on the phenotype of boys with autism, therefore, may lead to under-diagnosis of girls (see Attwood, 2007; Bishop, 2015; Hull et al., 2019; Lai et al., 2015; Rynkiewicz et al., 2016; Schuck et al., 2019). Here in the clinical settings under study, ambiguities about social behaviour can be explained without recourse to debates about how ‘masking’ might be separated from socialised behaviours more generally. In other words, clinicians are not tasked with examining a wider socio-political context where gender expectations inevitably shape behaviour.

In the autism assessment team, clinicians do not avoid or disregard uncertainty (Katz, 1984); the expression of doubt about the ADOS reinforces clinicians’ professionalism as those who understand the limitations of the tool (Pilnick and Zayts, 2014). We would suggest that expression of ambivalence and ambiguity in this setting is a means to deal interactionally with the inherent indeterminacy of the condition; to enable less experienced clinicians to raise conflicting issues; and ultimately achieve interactive progression towards collective accountability for the decision whilst maintaining a collegial atmosphere.

## 6 Conclusion

The assessment meeting allows for uncertainty, dispute, disagreement, ambivalence, co-existing conditions and a range of other complexities. This is a place where shared specialist knowledge of the contradictory and indeterminate nature of autism diagnosis provides ‘narrative scaffolding’ (Atkinson, 1995) for diagnostic deliberations, enabling progression towards diagnosis despite contradictory accounts.

In the process of assessment, therefore, clinicians contribute to the construction of autism as a biological entity rooted in the individual, and yet, at the same time, as a condition with symptoms that can be socially concealed (via masking, gender, under-reporting or co-conditions) or exaggerated (through over-reporting or rehearsing for assessment).

An individual, diagnostically, can only be autistic or not (Russell, 2014): we find that the achievement of this diagnostic binary is made possible through dispelling ambiguity and contradiction collectively in assessment meetings. We have shown how this work serves to counteract potential uncertainty, and utilises contemporary medical understandings of how autism can be ‘seen’ (and not seen) behaviourally within the clinic, and via patient and family reporting. Potential uncertainty caused by contradictory narratives is resolved through understanding that autism can be present but not seen (due to compensating or masking); seen but not present (due to behaviours that ‘look like’ autism, such as anxiety); or seen by some and not others (when patient and family member accounts conflict, for example). This resolution of contradiction from different aspects of the assessment then serves to create a narratively-coherent, intelligible clinical entity that is autism.

## Figures and Tables

**Table:** 

[ ]	Overlapping talk
,	Upward intonation
.	Falling intonation
↑	Marked pitch rise
::	Lengthening of preceding sound
£	Smiley voice
(.)	Micro pause
(0.2)	Timed pause (tenths of second)
> <	Speeded up talk
< >	Slowed down talk
So	Emphasis
=	‘Latching’ of successive talk
.hhh	In-breath
Bu-u-	Cut off of previous sound
°so°	Quieter speech
Bu**t**	Consonant produced more sharply than normal
	
Adapted from Hepburn and Bolden, 2013, Jefferson, 2004	

## Data statement

Access to the data that support these findings will be granted on request to the corresponding author via UK Data Service at http://reshare.ukdataservice.ac.uk/.

## Credit author statement

**Jennie Hayes:** Conceptualisation, Methodology, Project Administration, Investigation, Data Curation, Formal Analysis, Drafting – Original Draft Preparation; **Rose McCabe:** Supervision, Formal Analysis, Writing – reviewing and editing; **Tamsin Ford:** Supervision, Writing – reviewing and editing; **Daisy Parker**: Formal Analysis, Writing – reviewing and editing; **Ginny Russell:** Conceptualisation, Funding Acquisition, Supervision, Writing – reviewing and editing
